# Family History of Head and Neck Cancers

**DOI:** 10.3390/cancers13164115

**Published:** 2021-08-16

**Authors:** Xinjun Li, Anni I. Koskinen, Otto Hemminki, Asta Försti, Jan Sundquist, Kristina Sundquist, Kari Hemminki

**Affiliations:** 1 Center for Primary Health Care Research, Lund University, 20502 Malmö, Sweden; xinjun.li@med.lu.se (X.L.); a.foersti@dkfz.de (A.F.); jan.sundquist@med.lu.se (J.S.); kristina.sundquist@med.lu.se (K.S.); 2 Department of Otorhinolaryngology—Head and Neck Surgery, Helsinki University Hospital and University of Helsinki, 00029 Helsinki, Finland; anni.koskinen@helsinki.fi; 3 Department of Urology, Helsinki University Hospital and University of Helsinki, 00029 Helsinki, Finland; otto.hemminki@helsinki.fi; 4 Cancer Gene Therapy Group, Translational Immunology Research Program, University of Helsinki, 00029 Helsinki, Finland; 5 Hopp Children’s Cancer Center (KiTZ), 69120 Heidelberg, Germany; 6 Division of Pediatric Neurooncology, German Cancer Research Center (DKFZ), German Cancer Consortium (DKTK), 69120 Heidelberg, Germany; 7 Department of Family Medicine and Community Health, Department of Population Health Science and Policy, Icahn School of Medicine at Mount Sinai, New York, NY 10029, USA; 8 Center for Community-based Healthcare Research and Education (CoHRE), Department of Functional Pathology, School of Medicine, Shimane University, Izumo, Shimane 693-8501, Japan; 9 Faculty of Medicine and Biomedical Center in Pilsen, Charles University in Prague, 30605 Pilsen, Czech Republic; 10 Division of Cancer Epidemiology, German Cancer Research Centre (DKFZ), 69120 Heidelberg, Germany

**Keywords:** oral cancer, pharyngeal cancer, human papilloma virus, smoking, genetic factors

## Abstract

**Simple Summary:**

Head and neck cancers are cancers that arise between the mouth and larynx. Risk factors for these include smoking, alcohol, human papilloma virus (HPV) infection and family history. Because families can be identified for the whole Swedish population, we wanted to analyzed familial risks for HNC with same and different cancers among first-degree relatives. When a parent or sibling was diagnosed with HNC, other family members had a two-fold risk of being diagnosed with HNC, but the risk was higher when specific types of HNC, such as oral or nasopharyngeal cancers, were analyzed. Husbands of wives with cervical cancer had an increased risk of oropharyngeal cancer which may be related to shared HPV infection. In the Swedish population with low smoking levels, HPV is becoming a dominant risk factor, emphasizing the need for sexual hygiene and HPV vaccination.

**Abstract:**

Background: Head and neck cancers (HNCs) encompass a heterogeneous group of cancers between the mouth and larynx. Familial clustering in HNCs has been described, but how it influences individual sites and to which extent known risk factors, such as human papilloma virus (HPV) infection, may contribute is not well established. Patients/methods: We employed standardized incidence ratios (SIRs) to estimate familial risks for HNC with same (concordant) and different (discordant) cancers among first-degree relatives using data from the Swedish Cancer Registry from 1958 to 2018. Results: Incidence for male and female oropharyngeal cancer increased close to four-fold in the past 39 years. Familial HNC was found in 3.4% of the study population, with an overall familial SIR of 1.78. Patients with concordant nasopharyngeal cancer showed a high risk of 23.97, followed by hypopharyngeal cancer (5.43). The husbands of wives with cervical cancer had an increased risk of oropharyngeal cancer. Discussion/Conclusion: Nasopharyngeal cancers lacked associations with lifestyle or HPV associated cancers, suggesting a role for germline genetics, which was also true for the high-risk families of three HNC patients. In the Swedish population with low smoking levels, HPV is becoming a dominant risk factor, emphasizing the need for sexual hygiene and HPV vaccination.

## 1. Introduction

Head and neck cancers (HNCs) encompass a group of upper aerodigestive tract cancers often defined by squamous cell carcinoma (SCC) histology [1]. In Denmark, a neighboring country to Sweden, cancers in the larynx and oral cavity are the most common constituents of HNC, follow by oropharyngeal, hypopharyngeal, sinonasal, and nasopharyngeal cancers, with the male rates exceeding the female rates [2]. The incidence of HNC varies globally depending on risk factors; the incidence is increasing in many regions, whereby the estimated annual number of new diagnoses is reaching one million [1]. In Western countries, alcohol and tobacco and their interactions are the main risk factors, but chronic viral infections with human papillomavirus (HPV) and Epstein Barr virus (EBV) also contribute to the risk of HNC [3,4,5]. According to the International Agency for Research on Cancer (IARC), cancers of the pharynx and larynx are the most responsive sites, and those of the nasopharyx and nasal cavities are the least responsive sites for tobacco carcinogenesis [3]. According to the same source, risk reduction in HNCs after quitting is faster than what is known in lung cancer, and in 10 years of quitting, the risk of oral cavity and laryngeal cancers may reach the level of non-smokers. In global high-risk areas in the Indian subcontinent, betel quid chewing of areca nut products causes a high risk of cancer in the oral cavity, and in Southeast Asia, EBV and dietary habits contribute to endemic nasopharyngeal cancer risk [3,4]. The incidence of oropharyngeal and oral cancers has been increasing, particularly among men in Western countries during the recent decades, which has been associated with HPV infection and oral sex [2,6,7,8]. These cancers were also found in cervical cancer patients and their husbands, suggesting a shared HPV infection [9]. Oral HPV infection is shown to be the primary risk factor for HPV-related oropharyngeal cancer. Over 90% of oral HPV infections are sexually acquired [10]. HNCs are increased in immunocompromised individuals, such as kidney transplant patients, which may imply viral activation or another sensitivity to immune disturbance [11]. These mechanisms may also explain the high risk of HNCs as second primary cancers after a number of first primary cancers [12,13]. One special feature of HNCs is their presentation as multiple synchronous or metachronous primary tumors, a phenomenon known as “field cancerization” [1]. HPV-positive and -negative HNCs differ from each other in many ways, including prognosis [1,2]. Molecular control of HPV-positive HNCs is steered by the integrated HPV genome, which often leads to inactivation of p53 and other proteins controlling the cell cycle, while in HPV-negative cases, mutations of TP53 are common; survival is better in HPV-positive cases. Familial risks for HNC have been considered in several case-control and a few cohort studies, as discussed [5]. Large studies have been able to describe risks at individual sites, reporting clear effects of smoking, weaker effects of alcohol, but strong joint effects of both [14,15,16,17,18,19,20]. Oropharyngeal cancer is associated with cervical and lung cancers, suggesting the involvement of environmental risk factors [21]. In our earlier family studies, we usually considered HNC (upper aerodigestive tract) as an entity, because previously, the sample sizes for individual subsites were too small [6]. However, we have shown familial associations of HNC with lung and other smoking related cancers, including pancreas, stomach, and bladder cancers, and cancer of an unknown primary [6,22,23,24]. Another cluster of familial associations is with HPV-related anogenital cancers; for example, oropharyngeal and tonsillar cancers are strongly associated with in situ cervical cancer [25]. HNCs have been found in excess in individuals whose life situations may involve exposure to smoking, alcohol, and multiple sexual contacts, including the offspring of divorced parents, as well as individuals who have had children with many partners [26,27,28]. 

Germline genetic factors could influence the risk of HNC at many different levels, including the maintenance of DNA integrity against the DNA damaging influence of tobacco and alcohol [1]. The Fanconi anemia genes encode the enzymes that repair double-stand DNA breaks, and mutations in these genes cause a high risk of SCC in HNC in a rare syndrome [29,30]. Other rare cases may be related to P53 and other DNA repair gene mutations [1]. Lynch syndrome has been associated with HNC only in some single patient case reports. Acetaldehyde is the metabolite of ethanol, and its level is increased in individuals with a polymorphism in the aldehyde dehydrogenase (ALDH2) gene, resulting in a risk of HNC [3]. 

In the present study, we used the unique Swedish population data resources in order to assess familial clustering of HNC (diagnosed after age 20 years). It is noted that we had no individual data on important risk factors, such as smoking, alcohol, or HPV, but we had data on educational level, which, in part, may control for the lifestyle related risk factors. As HPV etiology is important in HNC, in addition to genetically related family members, we also included spouses in the analyses, with possible indicators of environmental etiology. The family relationships were obtained from a complete population register at Statistics Sweden, and all cancer data were derived from the Swedish Cancer Registry recently updated to the year 2018, guaranteeing the reliability of the data. 

## 2. Materials and Methods

For the Swedish Family-Cancer Database, family relationships were obtained from the Multigeneration Register, containing the Swedish population in families [31]. “The offspring generation” was born after 1931, and by 2018, the oldest offspring were 86 years old; siblings were only defined if they were in the offspring generation. Cancers were identified from the Swedish Cancer Registry, which was started in 1958 using codes of the International Classification of Diseases version 7 (and later 10). Accordingly, the codes were lip (140), tongue (141), mouth floor (143), other mouth (144), oropharynx (145), nasopharynx (146), hypopharynx (147), other pharynx (148), and larynx (161). Nasal cancer (160) was not included, as no concordant familial cancers were found. Information from the registers was linked at the individual level via the national 10-digit civic registration number. In the linked dataset, civic registration numbers were replaced with serial numbers so as to ensure anonymity.

Familial risk was considered for offspring whose first-degree relatives (parents or siblings) were diagnosed with the same (concordant) or a different (discordant) cancer; the relatives were thus probands. Standardized incidence ratios (SIRs) were calculated as the ratio of the observed to the expected number of cases. The expected numbers were calculated from the present dataset for all individuals without cancer in family members (i.e., most of the Swedish population), and the rates were standardized by five-year-age, gender, period (five-year groups), highest educational level (as proxy for socioeconomic status), and geographical region. The 95% confidence interval (95%CI) of the SIR was calculated assuming a Poisson distribution. The observed cases (O) indicated the persons for whom the SIR was calculated. For saving space in the busy tables, we did not show expected numbers, but they can be easily calculated by dividing O by SIR. Spouses were identified through the first common child of the couples.

Incidence data were obtained from the publicly available Nordcan database (https://www-dep.iarc.fr/NORDCAN/english/frame.asp, accessed on 15 June 2021), which is a compilation of data from the Nordic cancer registries, including the Swedish Cancer Registry, as described in [32]. Diagnosed patients of any age were included. The data were adjusted to the world standard population, and three-year moving averages were applied to smoothen the curves.

The study was approved by the Regional Ethical Review Board in Lund on 6 February 2013 (Reference 2012/795). Guidelines of the Helsinki Declaration were followed. The study was conducted in accordance with the approved guidelines, with explicit statement that no informed consent was required. The study is national register-based study on anonymous personal data.

## 3. Results

**Figure 1:**
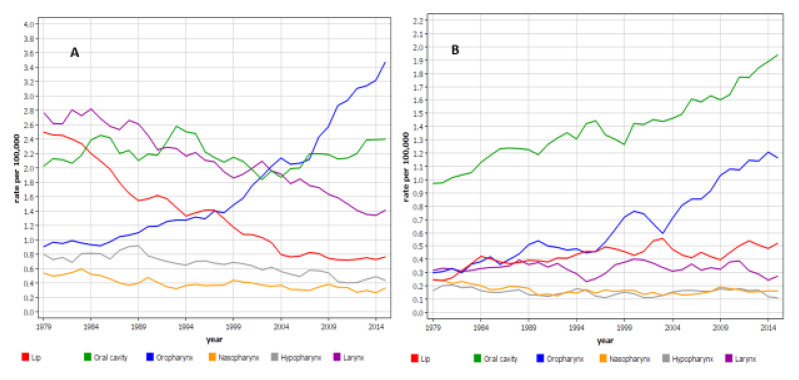
Age-standardized incidence per 100,000 in head and neck cancers in Sweden from 1978 to 2016 based on the Nordcan database for (**A**) men and (**B**) women. Note the difference in the scale for the y-axis. The curves are based on male/female patient numbers, which were 4636/1861 for lip, 6366/5201 for oral cavity, 4858/1925 for oropharynx, 964/460 for nasopharynx, 1936/529 for hypopharynx, and 6420/1047 for larynx. The data points were smoothened over 3 years.

Incidence rates for HNCs were analyzed from the Nordcan database (not from the Swedish Family-Cancer Database), spanning 39 years from 1978 to 2016 (Figure 1). The trends for incidence for most male sites were decreasing, for oral cavity the rate remained constant, but for oropharyngeal cancer there was a rate increase of more than 3.5 times. The female incidence rates were lower than the male rates, and for most sites they remained stable. The exceptions were oropharyngeal and oral cancers, with four-fold and two-fold increases, respectively.

The characteristics of the study population are described in Table S1. The total offspring generation index population at age 20 to 86 years amounted to 9.3 million individuals, recorded from 1933 to 31 December 2018. HNC was diagnosed in 12,617 individuals without a family history and in 450 individuals (3.4% of all) with a family history of HNC; the respective diagnostic ages were 58.4 and 58.7 years. The overall incidence (without family history) was 5.30/100,000 for men and 2.50/100,000 for women; for persons with a family history, the incidence rates were more than two times higher. The most common sites of HNC (without family history) were the oropharynx (23.6%) and tongue (22.4%).

### 3.1. Familial Risks for HNC

Familial risks for HNC according to the type and number of probands are shown in Table 1. When first-degree relatives were diagnosed with any HNC, the SIR was 1.78. SIRs were marginally higher for siblings than for offspring of affected parents, and for men compared with women. The SIR for the third male siblings was 126.05 when two siblings were already affected; these constituted two families in which two siblings were diagnosed with lip and other mouth cancer, and the third sibling was diagnosed with laryngeal cancer in one family and with hypopharyngeal cancer in the other. The SIR was 8.83 when two first-degree relatives were already affected in 20 families. Among the 60 recorded HNCs, the most common sites were tongue in 12 patients, other mouth in 10, hypopharynx in 10, larynx in 8, lip in 7, oropharynx in 6, and mouth floor in 4 patients.

The overall SIR for HNC was 1.05 (both sexes) when a family member was diagnosed with any cancer (Table 2). Among the discordant sites, male HNCs were increased when family members were diagnosed with esophageal (1.68), anal (1.60), lung (1.26), cervical (1.22), other female genital (1.61), bladder (1.14), and thyroid (1.43) cancers. SIR for female HNCs was increased when family members were diagnosed with liver (1.24) and lung (1.21) cancers.

### 3.2. Familial Risk for Site-Specific HNC

SIRs for HNCs are shown in Table 3 when family members were diagnosed by site-specific HNCs. The association was the highest for nasopharyngeal cancer (3.20), followed by cancers in the mouth floor (2.64) and hypopharynx (2.51). Although there were some differences between sexes, none of the SIRs differed significantly (i.e., 95%CIs overlapped).

Table 4 displays familial risk according to individual sites, and patients with concordant family history of nasopharyngeal cancer showed a high risk of 23.97. All of the concordant sites with case numbers showed significant associations, including hypopharynx (5.43). Among the discordant sites, high SIRs were noted between nasopharynx and hypopharynx (6.39), as well as between mouth floor and hypopharynx (7.45 and 8.35, respectively).

In Table 5, associations of the site specific HNCs are shown for any cancer. For simplicity, only sites with any significant associations are included. Lip cancer showed four associations, mouth floor one, oropharynx one, nasopharynx one, hypopharynx five, and larynx three; for tongue and other mouth, none were found. Esophageal cancer was associated with three HNC sites and lung with four; all other associations were with a single discordant cancer. Esophageal and lung cancers were associated with oropharyngeal, hypopharyngeal, and laryngeal cancers; for hypopharyngeal cancer the associations were high, and additionally included stomach and bladder cancers and non-Hodgkin lymphoma. Oropharyngeal cancer was associated with cervical cancer. The SIR for nasopharyngeal cancer was 4.24 when the family member was diagnosed with thyroid cancer.

### 3.3. Correlations between Spouses

An analysis of all or site-specific HNCs between spouses showed many SIRs higher than unity, but none that were significant (Table S2). However, when discordant cancers were analyzed, SIRs for the wives’ HNCs were increased when the husbands presented with rectal or lung cancers, or with cancer of an unknown primary (Table 6). SIRs for husbands’ HNCs were increased when their wives were diagnosed with lung or cervical cancers; the latter showing the highest risk of 1.57. In addition, other female genital cancer reached borderline significance (95%CI 0.98–1.96). When either spousal risks were combined, stomach cancer showed an additional association.

## 4. Discussion

The Swedish Family-Cancer Database is the largest family cancer database in the world, with premium features of nation-wide family structures and practically complete data on cancers from the high-quality cancer registry [31,33]. These features guarantee unbiased familial risk estimates, which may be difficult to achieve in case-control studies, sensitive to the selection of families and inaccurate reporting of cancers in family members. The nation-wide coverage over decades of registered cancers has enabled the collection of reasonable sample sizes even for a cancer for which familial cluster is rare, such as 3.4% for the present HNCs. We were able to identify 450 patients with a family history of HNC. This can be compared with the international consortium of 12 case-control studies with 305 familial patients; however, incidentally, a familial proportion of 3.6% was found, close to our estimate [16]. The novel aspects of the present study are the generation of concordant familial risks even for individual sites of HNCs, in addition to the discordant association with any cancer. As environmental and infectious agents are important risk factors for HNCs, the analysis of the risks between spouses was able to shed light on such non-genetic risk factors [1]. Our incidence data showed an increasing trend for oropharyngeal cancer in men and women, as well as for oral cancer in women [2,6,7,8]. Importantly, the increase in the female rate of oropharyngeal cancer equaled the male increase.

Among the novel familial risk estimates, Table 1 reports two families with three affected male siblings, with an SIR of 126.05; both families presented with lip and other mouth cancer, and additionally laryngeal cancer in one family and hypopharyngeal cancer in the other. Three patients with diverse HNCs were found in 10 families, with an SIR of 8.83. Concordant nasopharyngeal cancer showed an exquisitely high SIR of 23.97 (Table 4), followed by hypopharyngeal cancer of 5.43, and the remaining concordant sites of the lip, tongue, other mouth, oropharynx, and larynx ranged from 2.50 to 3.17. Mouth floor accounted for 4.5% of all HNCs and no concordant familial associations were found, but it showed a discordant association with cancers of the lip, tongue, other mouth, and hypopharynx. The above international consortium reported site-specific familial risks against all HNCs, and hypopharynx showed the highest risk of 2.28, while no data were given for nasopharynx [16]. Our similar analysis in Table 3 found an association for hypopharynx of 2.51, which ranked third after cancers of the nasopharynx (3.20) and mouth floor (2.64). The previous Swedish study, which covered cancers from 1959 to 2009, found an overall familial risk of 1.48 for HNC, and a high risk for female tongue cancer (6.37) and male pharyngeal cancer (3.13) among first-degree relatives [20]. The study from Utah reported four concordant patients with oropharyngeal cancer (estimated relative risk of 5.31) and three patients with hypopharyngeal cancer (relative risk of 19.17) [21]. They also reported associations of oropharyngeal cancer and oral and pharyngeal cancers.

Our analysis between HNCs and remote cancers revealed associations with lung cancer in men and women, and in women, associations with anal, cervical, and other female genital cancers. When site specific HNCs were considered, lung cancer association was the strongest with hypopharynx (2.21), and it was supported by high risks of other smoking related sites, namely the esophagus (4.34), stomach (1.54), and bladder (1.92). Association with cervical cancer was significant with oropharynx (1.47), consistent with HPV-related etiology. Other associations with indications for potential shared risk factors were clustering of skin melanoma and SCC with lip cancer (ultraviolet irradiation and possible immune disturbance). The association of non-Hodgkin lymphoma with hypopharyngeal cancer (1.91) could also signal immune disturbance. Interestingly, nasopharyngeal cancer with a high familial risk was not associated with any lifestyle related cancers. Its only significant association was with thyroid cancer. The meaning of this is not clear, but thyroid cancer was associated with any (male) HNCs, and examination of the thyroid is part of the diagnostic work-up for HNCs.

A significant spouse association with lung cancer was consistent with familial risk in cancers of the mouth floor, oropharynx, hypopharynx, and larynx, suggesting shared smoking habits as a risk factor. Husbands’ risk of HNCs was increased when wives had cervical and other female genital cancers. The association between cervical cancer and oropharyngeal cancer was also supported also by familial risks, implicating sexually transmitted HPV as a possible mechanism. According to our previous studies, husbands of cervical cancer and in-situ cervical cancer had an increased risk of many HNCs, including tonsils, as well as anal cancer [9,34]. The recent literature has emphasized male risk of oropharyngeal cancer, including those related to HPV-infection [1,7]. We have no data on HPV status in our population, but a large study in the Stockholm area, which documented an increase in oropharyngeal cancer incidence, reported HPV positivity at 70%, slightly higher for men than for women (72 vs. 64%) and a marginal increase from 67 to 70% over the period from 2000 to 2016 [8]. The equally high relative increase in oropharyngeal cancer in men and women (Figure 1) does not support particular male susceptibility. The other notable increase in women was that of oral cavity, which has also been noted in international studies [7]. When HPV infection is sexually transmitted, the largest reservoir of infected patients is found among cervical in situ patients, followed by invasive cervical cancer patients [9,35]. Second primary cancers are common between HPV related sites, including oropharyngeal and oral cancers after cervical or anal cancers [35,36,37,38]. In a meta-analysis, including our Swedish studies, the risk of second oropharyngeal cancer was associated with first cancers of the cervix (1.61), cervix in situ (2.40), vulva−vagina (5.95), anus (1.75), penis (3.88), and oropharynx (22.45) [39]. Considering that in situ cervical cancer is by far the most common cancer among those listed, it is likely that women are infected at oropharyngeal and other HNC sites from the primary sites, which may be the cause for the high increase in female incidence in oropharyngeal and oral cancers. Such data may indicate that even non-sexual contacts, such as kissing, may transmit HPV-infection. Oral HPV prevalence was in around 10% in Swedish young men and women before the national HPV vaccination was started in 2012 [40]. Cervical samples were HPV positive in over 70% of these young women. According to a Finnish study, 17% of the oral samples from young women tested HPV positive, and 18.7% of their spouses were also positive [41].

The main limitations of the study are the lack of information on risk factors and the descriptive nature of the results confined to the population under study. Most causes for familial clustering are unknown, even though circumstantial evidence suggests the involvement of putative risk factors. Smoking is an important risk factor, which was consistent with increased risks in many smoking-related sites despite adjustment for educational level (i.e., socioeconomic factors). Educational level is highly correlated with lung cancer risk in Sweden, accounting for a population attributable fraction of 38% [42]. Another factor influencing confounding by smoking is that the prevalence of smoking in Swedish men has been the lowest in Europe, at least since 1980 [43,44]. Because of the low background level of smoking, the role of HPV infections has become more prominent.

In summary, we showed that the first-degree familial clustering of HNC was modest, accounting for 3.4% of patients. Very high familial risks (24-fold) were observed for concordant nasopharyngeal cancer, and this cancer also showed the highest familial association with any HNC. Nasopharyngeal cancer lacked discordant associations with lifestyle or HPV associated sites, suggesting a role for germline genetics and DNA damage repair [29,30]. The high risks that were observed in the families of three HNC patients showed clustering of lip, mouth, tongue, and other cancers. There was no indication that these HNC clusters were biased towards lifestyle or HPV-related cancers, proposing a role for genetic interactions, possibly in combination with immune dysfunction. The clinical take home message is that while the old risk factors of smoking and alcohol are still valid and targets for prevention, HPV is likely becoming the dominant risk factor in the Swedish population with a low smoking level.

It is urgently important to understand the routes of transmission of the HPV infections related to HNCs. While concerns have been focused on increasing incidence of HPV-related oropharyngeal cancers in men, our incidence data show that the relative increase in oropharyngeal cancer was equally high in women. It is important to create greater public awareness of HPV positive oropharyngeal cancer and preventative measures to avoid HPV infection. Widespread use of vaccines will hopefully help to turn down the growing incidence curve, and for those already at risk due to prior exposure, the development of effective screening techniques will be crucial in order to enable the early detection of subclinical lesions. Good news is that, in the HPV vaccinated population, the prevalence of HPV positive oral and cervical samples dropped to a low level for the HPV types targeted in the vaccine [40].

## Figures and Tables

**Table 1:** Familial risk of head and neck cancers when first-degree relatives were diagnosed with head and neck cancers.

Proband	Men				Women				All			
	O	SIR	95% CI		O	SIR	95% CI		O	SIR	95% CI	
Family history (any first-degree relatives)	315	**1.81**	**1.61**	**2.02**	135	**1.71**	**1.43**	**2.02**	450	**1.78**	**1.62**	**1.95**
Parents	190	**1.68**	**1.45**	**1.93**	92	**1.77**	**1.43**	**2.17**	282	**1.71**	**1.51**	**1.92**
Both parents	1	3.23	0.00	18.52	1	8.64	0.00	49.54	2	4.70	0.44	17.29
Father	142	**1.69**	**1.42**	**1.99**	69	**1.81**	**1.41**	**2.29**	211	**1.73**	**1.50**	**1.98**
Mother	49	**1.59**	**1.18**	**2.11**	24	**1.70**	**1.09**	**2.54**	93	**1.78**	**1.44**	**2.18**
Siblings	135	**2.07**	**1.74**	**2.45**	45	**1.54**	**1.12**	**2.06**	180	**1.91**	**1.64**	**2.21**
One sibling	129	**1.96**	**1.63**	**2.33**	45	**1.55**	**1.13**	**2.07**	174	**1.83**	**1.57**	**2.13**
Two siblings	6	**126.05**	**45.37**	**276.18**	0				6	**98.85**	**35.57**	**216.57**
One family member	298	**1.73**	**1.54**	**1.93**	132	**1.69**	**1.41**	**2.00**	430	**1.71**	**1.56**	**1.88**
Two family members	34	**11.23**	**6.53**	**18.02**	6	3.99	0.75	11.82	40	**8.83**	**5.38**	**13.66**

O = observed, SIR = standardized incidence ratio, CI = confidence intervals. Bold values indicate that the 95% CI does not include 1.00.

**Table 2:** Familial risk of head and neck cancers when family members were diagnosed with any cancer.

Cancer in Family	Men				Women				All			
	O	SIR	95% CI		O	SIR	95% CI		O	SIR	95% CI	
Head and neck	315	**1.81**	**1.61**	**2.02**	135	**1.71**	**1.43**	**2.02**	450	**1.78**	**1.62**	**1.95**
Salivary gland	20	1.09	0.67	1.69	10	1.27	0.61	2.35	30	1.15	0.77	1.64
Esophagus	108	**1.68**	**1.37**	**2.02**	29	1.01	0.67	1.45	137	**1.47**	**1.23**	**1.74**
Stomach	267	1.00	0.88	1.13	117	0.99	0.82	1.19	384	1.00	0.90	1.10
Small intestine	35	1.00	0.69	1.39	13	0.85	0.45	1.46	48	0.95	0.70	1.26
Colon	558	1.02	0.94	1.11	217	0.89	0.78	1.02	775	0.98	0.91	1.05
Rectum	301	1.07	0.95	1.20	120	0.99	0.83	1.18	421	1.03	0.94	1.14
Anus	25	**1.60**	**1.04**	**2.37**	12	1.75	0.90	3.07	37	**1.65**	**1.16**	**2.27**
Liver	169	0.97	0.83	1.12	97	**1.24**	**1.01**	**1.52**	266	1.05	0.93	1.19
Pancreas	177	1.01	0.86	1.17	85	1.08	0.86	1.34	262	1.03	0.91	1.16
Lung	605	**1.26**	**1.16**	**1.37**	260	**1.21**	**1.06**	**1.36**	865	**1.24**	**1.16**	**1.33**
Breast	724	0.95	0.89	1.03	339	1.00	0.89	1.11	1063	0.97	0.91	1.03
Cervix	114	**1.22**	**1.00**	**1.46**	48	1.13	0.83	1.49	162	**1.19**	**1.01**	**1.39**
Endometrium	180	1.03	0.88	1.19	66	0.85	0.66	1.09	246	0.97	0.86	1.10
Ovary	124	1.05	0.87	1.25	47	0.90	0.66	1.20	171	1.01	0.86	1.17
Other female genitals	40	**1.61**	**1.15**	**2.19**	7	0.62	0.25	1.29	47	1.30	0.96	1.73
Prostate	711	0.89	0.83	0.96	371	1.05	0.95	1.16	1082	0.94	0.88	1.00
Testis	12	0.90	0.47	1.59	12	1.81	0.93	3.16	24	1.21	0.77	1.80
Other male genitals	10	1.10	0.53	2.04	8	2.02	0.86	4.01	18	1.38	0.82	2.19
Kidney	144	1.14	0.96	1.34	54	0.94	0.70	1.22	198	1.07	0.93	1.23
Bladder	242	**1.14**	**1.00**	**1.29**	101	1.06	0.86	1.29	343	**1.11**	**1.00**	**1.24**
Melanoma	149	0.99	0.84	1.16	71	1.02	0.80	1.29	220	1.00	0.87	1.14
Skin	227	1.13	0.99	1.29	93	1.03	0.83	1.26	320	1.10	0.98	1.23
Eye	9	0.82	0.37	1.56	3	0.58	0.11	1.73	12	0.74	0.38	1.30
Nervous system	94	0.84	0.68	1.03	43	0.85	0.62	1.15	137	0.85	0.71	1.00
Thyroid	41	**1.43**	**1.02**	**1.94**	14	1.06	0.57	1.78	55	1.31	0.99	1.70
Endocrine	67	1.05	0.81	1.34	31	1.07	0.73	1.52	98	1.06	0.86	1.29
Bone	2	0.36	0.03	1.31	3	1.18	0.22	3.49	5	0.61	0.19	1.44
Connective tissue	13	0.55	0.29	0.95	9	0.87	0.39	1.65	22	0.65	0.41	0.98
Primary unknown	118	1.11	0.92	1.33	58	1.20	0.91	1.55	176	1.14	0.97	1.32
Hodgkins disease	17	1.04	0.60	1.66	6	0.83	0.30	1.81	23	0.97	0.62	1.46
Non-Hodgkins lymphoma	126	1.10	0.92	1.31	53	1.03	0.77	1.35	179	1.08	0.93	1.25
Myeloma	54	1.03	0.77	1.35	31	1.31	0.89	1.87	85	1.12	0.89	1.38
Leukemia	90	0.77	0.62	0.95	49	0.94	0.69	1.24	139	0.82	0.69	0.97
Others	8	0.94	0.40	1.86	2	0.53	0.05	1.97	10	0.81	0.39	1.50
All	5896	**1.06**	**1.03**	**1.08**	2614	**1.05**	**1.01**	**1.09**	8510	**1.05**	**1.03**	**1.08**

O = observed, SIR = standardized incidence ratio, CI = confidence intervals. Bold values indicate that the 95% CI does not include 1.00.

**Table 3:** Familial risk of site-specific head and neck cancers when first-degree relatives were diagnosed with head and neck cancers.

Subtypes of Head and Neck Cancer in the Study Population	Men				Women				All			
	O	SIR	95% CI		O	SIR	95% CI		O	SIR	95% CI	
Lip	23	1.50	0.95	2.25	20	**2.18**	**1.33**	**3.37**	43	**1.75**	**1.27**	**2.36**
Tongue	57	**1.60**	**1.21**	**2.07**	26	1.15	0.75	1.69	83	**1.43**	**1.14**	**1.77**
Mouth floor	21	**2.64**	**1.63**	**4.04**	12	**2.64**	**1.36**	**4.63**	33	**2.64**	**1.82**	**3.71**
Other mouth	40	**2.02**	**1.44**	**2.75**	18	1.20	0.71	1.89	58	**1.66**	**1.26**	**2.15**
Oropharynx	71	**1.60**	**1.25**	**2.02**	28	**1.81**	**1.20**	**2.62**	99	**1.66**	**1.35**	**2.02**
Nasopharynx	20	**3.13**	**1.91**	**4.84**	8	**3.42**	**1.46**	**6.76**	28	**3.20**	**2.13**	**4.64**
Hypopharynx	21	**2.37**	**1.46**	**3.63**	7	**3.04**	**1.20**	**6.29**	28	**2.51**	**1.66**	**3.63**
Other pharynx	2	2.77	0.26	10.18	0				2	1.71	0.16	6.29
Larynx	60	**1.71**	**1.30**	**2.20**	16	**2.25**	**1.28**	**3.67**	76	**1.80**	**1.42**	**2.25**

O = observed, SIR = standardized incidence ratio, CI = confidence intervals. Bold values indicate that the 95% CI does not include 1.00.

**Table 4:** Familial risk of site-specific head and neck cancers when family members were diagnosed with site specific head and neck cancers.

Cancer in Family	Lip				Tonge				Mouth Floor				Other Mounth			
	O	SIR	95% CI		O	SIR	95% CI		O	SIR	95% CI		O	SIR	95% CI	
Lip	14	**2.50**	**1.36**	**4.21**	14	1.17	0.64	1.97	7	**2.63**	**1.04**	**5.45**	11	1.48	0.73	2.65
Tongue	6	1.80	0.65	3.94	27	**3.17**	**2.09**	**4.62**	6	**3.43**	**1.24**	**7.52**	12	**2.43**	**1.25**	**4.26**
Mouth floor	4	**3.98**	**1.04**	**10.29**	5	1.95	0.61	4.58	0				1	0.67	0.00	3.85
Other mouth	6	1.69	0.61	3.70	13	1.54	0.82	2.64	6	**3.34**	**1.20**	**7.32**	14	**2.75**	**1.50**	**4.63**
Oropharynx	1	0.37	0.00	2.09	9	1.24	0.56	2.36	2	1.33	0.13	4.90	7	1.69	0.67	3.50
Nasopharynx	2	1.88	0.18	6.92	0				1	1.87	0.00	10.73	0			
Hypopharynx	3	1.91	0.36	5.64	6	1.66	0.60	3.65	6	**7.45**	**2.68**	**16.33**	2	0.91	0.09	3.36
Larynx	7	1.26	0.50	2.62	9	0.69	0.31	1.31	5	1.75	0.55	4.13	10	1.27	0.61	2.35
All	43	**1.75**	**1.27**	**2.36**	83	**1.43**	**1.14**	**1.77**	33	**2.64**	**1.82**	**3.71**	58	**1.66**	**1.26**	**2.15**
**Cancer in family**	**Oropharynyx**				**Nasopharynx**				**Hypopharynx**				**Larynx**			
	**O**	**SIR**	**95% CI**		**O**	**SIR**	**95% CI**		**O**	**SIR**	**95% CI**		**O**	**SIR**	**95% CI**	
Lip	12	0.97	0.50	1.71	4	2.33	0.61	6.03	2	0.82	0.08	3.02	11	1.17	0.58	2.10
Tongue	13	1.52	0.81	2.61	2	1.55	0.15	5.69	4	2.63	0.68	6.80	9	1.56	0.71	2.97
Mouth floor	7	**2.60**	**1.03**	**5.38**	2	5.10	0.48	18.75	4	**8.35**	**2.17**	**21.60**	5	2.84	0.90	6.69
Other mouth	9	1.06	0.48	2.01	2	1.62	0.15	5.97	0				8	1.31	0.56	2.58
Oropharynx	19	**2.54**	**1.53**	**3.97**	3	2.55	0.48	7.55	4	3.12	0.81	8.06	12	**2.49**	**1.28**	**4.36**
Nasopharynx	4	1.59	0.41	4.11	9	**23.97**	**10.87**	**45.71**	3	**6.39**	**1.21**	**18.93**	4	2.18	0.57	5.65
Hypopharynx	8	2.13	0.91	4.22	2	3.67	0.35	13.48	4	**5.43**	1.41	14.03	1	0.36	0.00	2.09
Larynx	26	**1.91**	**1.25**	**2.80**	4	2.03	0.53	5.26	7	**2.73**	**1.08**	**5.66**	26	**2.73**	**1.78**	**4.00**
All	99	**1.66**	**1.35**	**2.02**	28	**3.20**	**2.13**	**4.64**	28	**2.51**	**1.66**	**3.63**	76	**1.80**	**1.42**	**2.25**

O = observed, SIR = standardized incidence ratio, CI = confidence intervals. Bold values indicate that the 95% CI does not include 1.00.

**Table 5:** Familial risk of site-specific head and neck cancers when family members were diagnosed with any cancer.

Cancer in Family	Lip				Tongue				Mouth Floor				Other Mounth			
	O	SIR	95% CI		O	SIR	95% CI		O	SIR	95% CI		O	SIR	95% CI	
Esophagus	9	1.00	0.45	1.90	24	1.12	0.72	1.67	9	1.94	0.88	3.70	11	0.86	0.43	1.54
Stomach	48	1.19	0.88	1.58	67	0.78	0.60	0.99	24	1.26	0.81	1.88	49	0.90	0.67	1.19
Colon	84	1.10	0.88	1.36	160	0.87	0.74	1.01	31	0.81	0.55	1.14	102	0.93	0.76	1.13
Liver	36	**1.44**	**1.01**	**2.00**	53	0.92	0.69	1.20	11	0.88	0.44	1.59	36	1.02	0.72	1.42
Lung	68	1.04	0.81	1.32	182	1.13	0.97	1.30	50	**1.47**	**1.09**	**1.94**	98	1.03	0.84	1.26
Cervix	15	1.19	0.66	1.96	22	0.69	0.43	1.05	12	1.80	0.92	3.15	20	1.08	0.66	1.67
Bladder	38	1.33	0.94	1.82	74	1.02	0.80	1.28	14	0.94	0.51	1.59	37	0.87	0.61	1.20
Melanoma	29	**1.54**	**1.03**	**2.21**	50	0.91	0.68	1.20	7	0.68	0.27	1.41	32	1.05	0.72	1.48
Skin	58	**2.18**	**1.66**	**2.82**	72	1.03	0.81	1.30	15	1.08	0.60	1.78	44	1.09	0.79	1.47
Thyroid	7	1.84	0.73	3.82	13	1.27	0.68	2.18	3	1.51	0.28	4.47	8	1.36	0.58	2.69
Primary unknown	25	**1.70**	**1.10**	**2.51**	29	0.80	0.53	1.15	6	0.80	0.29	1.75	26	1.21	0.79	1.77
Non-Hodgkins lymphoma	17	1.12	0.65	1.79	41	1.02	0.73	1.39	6	0.76	0.27	1.66	23	1.00	0.63	1.50
Myeloma	10	1.39	0.66	2.56	19	1.05	0.63	1.65	2	0.55	0.05	2.01	11	1.03	0.51	1.85
All	913	**1.20**	**1.12**	**1.28**	1802	0.95	0.90	0.99	411	1.05	0.95	1.16	1102	0.98	0.93	1.04
**Cancer in family**	**Oropharynx**				**Nasopharynx**				**Hypopharynx**				**Larynx**			
	**O**	**SIR**	**95% CI**		**O**	**SIR**	**95% CI**		**O**	**SIR**	**95% CI**		**O**	**SIR**	**95% CI**	
Esophagus	36	**1.64**	**1.15**	**2.27**	1	0.31	0.00	1.80	18	**4.34**	**2.57**	**6.88**	28	**1.79**	**1.19**	**2.59**
Stomach	76	0.88	0.70	1.11	16	1.32	0.75	2.15	27	**1.54**	**1.02**	**2.25**	74	1.09	0.86	1.37
Colon	183	0.98	0.84	1.13	21	0.79	0.49	1.21	33	0.97	0.67	1.36	157	**1.20**	**1.02**	**1.40**
Liver	67	1.14	0.89	1.45	8	0.95	0.40	1.87	12	1.07	0.55	1.88	42	0.98	0.71	1.33
Lung	201	**1.20**	**1.04**	**1.38**	32	1.30	0.89	1.84	67	**2.21**	**1.71**	**2.80**	164	**1.44**	**1.23**	**1.68**
Cervix	48	**1.47**	**1.08**	**1.95**	5	1.00	0.31	2.35	10	1.70	0.81	3.13	30	1.34	0.91	1.92
Bladder	83	1.12	0.89	1.39	18	1.66	0.98	2.64	25	**1.92**	**1.24**	**2.84**	54	1.09	0.82	1.42
Melanoma	51	0.94	0.70	1.24	10	1.15	0.55	2.13	13	1.51	0.80	2.58	27	0.82	0.54	1.19
Skin	68	0.96	0.74	1.22	9	0.89	0.41	1.71	11	0.91	0.45	1.64	43	0.94	0.68	1.26
Thyroid	9	0.90	0.41	1.72	7	**4.24**	**1.68**	**8.78**	1	0.59	0.00	3.41	7	1.05	0.42	2.18
Primary unknown	44	1.20	0.87	1.61	9	1.67	0.76	3.18	10	1.53	0.73	2.82	27	1.06	0.70	1.55
Non-Hodgkins lymphoma	45	1.13	0.83	1.52	4	0.65	0.17	1.69	13	**1.91**	**1.01**	**3.27**	29	1.10	0.74	1.58
Myeloma	29	**1.62**	**1.08**	**2.33**	4	1.51	0.39	3.91	3	0.95	0.18	2.80	7	0.57	0.22	1.17
All	2039	**1.06**	**1.02**	**1.11**	330	**1.16**	**1.04**	**1.30**	472	**1.38**	**1.26**	**1.51**	1400	**1.06**	**1.01**	**1.12**

O = observed, SIR = standardized incidence ratio, CI = confidence intervals. Bold values indicate that the 95% CI does not include 1.00.

**Table 6:** Risk of head and neck cancers in a spouse when the other spouse was diagnosed with any cancer.

Cancer in Spouse	Head and Neck Cancer in Wives				Head and Neck Cancer in Husbands				Head and Neck Cancer in Spouses			
	O	SIR	95% CI		O	SIR	95% CI		O	SIR	95% CI	
Head and neck	71	1.12	0.88	1.42	46	1.17	0.85	1.56	117	1.14	0.94	1.37
Salivary gland	6	1.23	0.44	2.69	7	1.03	0.41	2.14	13	1.11	0.59	1.91
Esophagus	26	1.03	0.67	1.51	19	1.50	0.90	2.35	45	1.19	0.87	1.59
Stomach	87	1.23	0.98	1.51	66	1.21	0.94	1.55	153	**1.22**	**1.04**	**1.43**
Small intestine	9	0.81	0.37	1.54	15	1.09	0.61	1.80	24	0.96	0.62	1.44
Colon	159	1.01	0.86	1.17	234	0.97	0.85	1.11	393	0.99	0.89	1.09
Rectum	136	**1.23**	**1.08**	**1.40**	98	0.94	0.76	1.14	234	1.12	0.99	1.28
Anus	4	1.41	0.37	3.64	14	1.27	0.69	2.14	18	1.30	0.77	2.06
Liver	61	1.26	0.97	1.62	67	0.99	0.77	1.26	128	1.10	0.92	1.31
Pancreas	64	1.22	0.94	1.55	76	1.05	0.82	1.31	140	1.12	0.94	1.32
Lung	246	**1.23**	**1.08**	**1.40**	286	**1.38**	**1.22**	**1.55**	532	**1.31**	**1.20**	**1.42**
Breast	1	0.30	0.00	1.72	984	1.03	0.96	1.09	985	1.03	0.96	1.09
Cervix	0				156	**1.57**	**1.34**	**1.84**	156	**1.57**	**1.34**	**1.84**
Endometrium	0				199	0.92	0.80	1.06	199	0.92	0.80	1.06
Ovary	0				138	0.99	0.83	1.17	138	0.99	0.83	1.17
Other female genitals	0				35	1.41	0.98	1.96	35	1.41	0.98	1.96
Prostate	721	0.95	0.89	1.03	0				721	0.95	0.89	1.03
Testis	11	0.86	0.43	1.54	0				11	0.86	0.43	1.54
Other male genitals	6	0.73	0.26	1.61	0				6	0.73	0.26	1.61
Kidney	61	0.88	0.67	1.13	56	0.84	0.64	1.09	117	0.86	0.71	1.03
Bladder	176	1.06	0.91	1.23	81	1.09	0.87	1.36	257	1.07	0.94	1.21
Melanoma	76	0.81	0.64	1.01	128	0.86	0.72	1.02	204	0.84	0.73	0.96
Skin	131	0.98	0.82	1.16	132	0.88	0.73	1.04	263	0.92	0.82	1.04
Eye	5	0.80	0.25	1.88	2	0.23	0.02	0.86	7	0.47	0.19	0.98
Nervous system	43	0.75	0.54	1.01	119	1.12	0.93	1.35	162	0.99	0.85	1.16
Thyroid	10	1.06	0.51	1.96	46	1.22	0.89	1.62	56	1.19	0.90	1.54
Endocrine	19	0.74	0.44	1.15	70	0.88	0.68	1.11	89	0.84	0.68	1.04
Bone	6	1.85	0.67	4.06	3	0.81	0.15	2.39	9	1.29	0.59	2.47
Connective tissue	21	1.49	0.92	2.27	14	0.78	0.42	1.31	35	1.09	0.76	1.51
Hodgkins disease	3	0.32	0.06	0.95	8	0.84	0.36	1.66	11	0.58	0.29	1.05
Non-Hodgkins lymphoma	63	0.81	0.62	1.04	110	1.19	0.98	1.43	173	1.02	0.87	1.18
Myeloma	40	1.18	0.84	1.60	47	1.25	0.91	1.66	87	1.21	0.97	1.50
Leukemia	69	0.87	0.67	1.10	91	0.96	0.77	1.18	160	0.92	0.78	1.07
Primary unknown	76	**1.37**	**1.08**	**1.71**	113	1.14	0.94	1.37	189	**1.22**	**1.05**	**1.41**
All	2435	**1.05**	**1.00**	**1.09**	3476	**1.06**	**1.03**	**1.08**	5911	**1.05**	**1.03**	**1.08**

O = observed, SIR = standardized incidence ratio, CI = confidence intervals. Bold values indicate that the 95% CI does not include 1.00.

## Data Availability

Data are used under a specific agreement between K.S, J.S. and the Swedish Board of Health and Welfare. Anyone wanting to use these data should contact the Swedish Board.

## Supplementary Materials

The following are available online at https://www.mdpi.com/article/10.3390/cancers13164115/s1, Table S1: Characteristics of the study population of head and neck cancer with and without family history of head and neck cancer, 1958–2018, Table S2: Risks of head and neck cancer among spouses.
