# Supplementary material for: Family History of Head and Neck Cancers
Source: Cancers (Basel). 2021 Aug 16;13(16):4115. doi: 10.3390/cancers13164115 (PMC8392405; doi:10.3390/cancers13164115)
Supplement: Supplementary file 1 [file cancers-13-04115-s001.zip › cancers-1292324-supplementary.pdf]

cancers MDPI

Table S1. Characteristics of the study population of head and neck cancer with and without family history of head and neck cancer, 1958–2018.

Definition	With Family History of Head and Neck Cancer		Non family History of Head and Neck Cancer	
	No.	%	No.	%
**Population (*n* = 9,338,882)**	92117	100.0	9,246,765	100.0
Men (*n* = 4,792,416)	47033	51.1	4,745,383	51.3
Women (*n* = 4,546,466)	45084	48.9	4,501,382	48.7
**Total case of head and neck cancer**				
n. of case (*n* = 13,067) (% of all cases)	450	3.4	12617	96.6
Mean age at diagnosis (± SD)	58.7 ± 10.7		58.4 ± 12.2	
Incidence rate (per 100 000 person years), 95% CI	9.77, 8.86–10.67		3.93, 3.86–4.00	
**Men**				
n. of case (*n* = 9007)	315	70.0	8692	68.9
Mean age at diagnosis (± SD)	58.9 ± 9.9		58.6 ± 11.6	
Incidence rate (per 100 000 person years), 95% CI	13.49, 12.00–14.99		5.30, 5.19–5.41	
**Women**				
n. of case (*n* = 4060)	135	30.0	3925	31.1
Mean age at diagnosis (± SD)	58.1 ± 12.5		57.8 ± 13.6	
Incidence rate (per 100 000 person years), 95% CI	5.94, 4.94–6.94		2.50, 2.42–2.58	
**Subtypes of head and neck cancer (*n* = 13,067)**	450	100.0	12617	100.0
Lip (ICD-7 140, *n* = 1331)	43	9.6	1288	10.2
Tongue (ICD-7 141, *n* = 2915)	83	18.4	2832	22.4
Mouth floor (ICD7 143, *n* = 622)	33	7.3	589	4.7
Other mouth (ICD-7 144, *n* = 1752)	58	12.9	1694	13.4
Oropharynx (ICD-7 145, *n* = 3082)	99	22.0	2983	23.6
Nasopharynx (ICD-7 146, *n* = 532)	28	6.2	504	4.0
Hypopharynx (ICD-7 147, *n* = 655)	28	6.2	627	5.0
Other pharynx (ICD-7 148, *n* = 62)	2	0.4	60	0.5
Larynx (ICD-7 161, *n* = 2116)	76	16.9	2040	16.2

CI: Confidence interval, SD: Standardized deviation.

Table S2. Risks of head and neck cancer among spouses.

Subtypes of Head and Neck Cancer in Spouse	Head and neck Cancer in Wives				Head and Neck Cancer in Husbands				Head and Neck Cancer in Spouses			
	O	SIR	95% CI		O	SIR	95% CI		O	SIR	95% CI	
Lip	17	1.40	0.82	2.25	6	0.93	0.33	2.03	23	1.24	0.78	1.86
Tongue	6	0.71	0.26	1.56	13	1.53	0.81	2.62	19	1.12	0.67	1.76
Mouth floor	5	1.72	0.54	4.05	3	1.41	0.27	4.17	8	1.59	0.68	3.14
Other mouth	5	0.74	0.23	1.74	7	0.79	0.31	1.64	12	0.77	0.40	1.35
Oropharynx	11	1.31	0.65	2.35	6	0.97	0.35	2.13	17	1.17	0.68	1.87
Nasopharynx	4	1.44	0.38	3.73	3	1.98	0.37	5.87	7	1.63	0.65	3.39
Hypopharynx	6	1.43	0.52	3.14	1	0.62	0.00	3.55	7	1.21	0.48	2.50
Larynx	17	0.98	0.57	1.57	7	1.70	0.67	3.52	24	1.12	0.72	1.67
All	71	1.12	0.88	1.42	46	1.17	0.85	1.56	117	1.14	0.94	1.37

O = Observed, SIR = Standardized incidence ratio, CI = Confidence intervals.
